# Sonic Hedgehog signaling in spinal cord injury: mechanisms and therapeutic implications

**DOI:** 10.3389/fnmol.2025.1624501

**Published:** 2025-07-30

**Authors:** Mingjuan Du, Xiaozhen Ji, Weiting Chen

**Affiliations:** ^1^Department of Emergency, Taizhou Integrated Chinese and Western Medicine Hospital, Taizhou, Zhejiang, China; ^2^Longquan People’s Hospital, Lishui, China; ^3^The First People’s Hospital of Linhai, Taizhou, China

**Keywords:** Gli transcription factors, neuron, SMO, Sonic Hedgehog signaling pathway, spinal cord injury

## Abstract

Spinal cord injury is a serious neurological condition that results in severe dysfunction below the level of injury, often leading to long-term disability and impaired quality of life. Despite significant advances in medical care, effective treatment options remain limited. Recent research has highlighted the role of endogenous signaling pathways, including Sonic Hedgehog, in the natural healing response following SCI. Sonic Hedgehog (Shh) signaling pathway plays a vital role in spinal cord development and post-injury regeneration by regulating neuroprotection, axon regeneration, synaptic remodeling and inflammation. Shh exerts its effects through a well-defined cascade involving Patched (Ptch), Smoothened (Smo) and Gli transcription factors, ultimately influencing genes involved in neural repair. Various pharmacological agents including agonists (SAG, Purmorphamine and Shh-N) and antagonists (Cyclopamine, Vismodegib and Sonidegib) have been studied for their ability to modulate this pathway and enhance recovery in preclinical models. In addition, emerging approaches such as stem cell therapies, exosome delivery and nanotechnology-based drug targeting are under investigation to improve the efficacy and specificity of Shh-based treatments. However, despite promising experimental outcomes, the clinical translation of these findings faces significant challenges, including delivery limitations, potential tumorigenicity, immune variability and the lack of robust human data. This review critically examines the molecular mechanisms and therapeutic potential of Shh signaling in SCI, highlights current limitations and conflicting evidence and outlines future directions to bridge the gap between preclinical findings and clinical application.

## 1 Introduction

Spinal Cord Injury is a severe, high-impact condition that causes significant damage to the central nervous system, often resulting in severe dysfunction in motor, sensory and autonomic functions below the affected site (Conti et al., 2023). SCI not only leads to substantial physical and psychological impairment for the patient, but also imposes a huge economic burden on society (Conti et al., 2023). SCI can occur in both traumatic and non-traumatic forms. Traumatic SCI is the most commonly reported injuries worldwide due to sudden, forceful impact or trauma to the spine, that damages the spinal cord and disrupts neural communication (Hu et al., 2023). The annual incidence rate of traumatic SCI ranges from 10.4 to 83 cases per million globally, varying byregion and economic development (Conti et al., 2023; Wu et al., 2022). For example in China, the incidence of traumatic SCI increased from 45.1 cases per million in 2009 to 66.5 cases per million in 2018 (Hao et al., 2021).

Patho-physiologically, SCI comprises both primary and secondary phases of injury (Ahuja et al., 2017). The primary injury involves the immediate mechanical damage at site of injury which may result from compression, laceration or stretching of the spinal cord. The secondary injury follows this initial trauma and involves complex processes such as edema, hypotension, hypoxemia and hemorrhage that exacerbate tissue damage (Patek and Stewart, 2023).

Even though, there are significant improvements in the field of medical science, the modern clinical practice provides an insufficient model of spinal cord injury (SCI) management. The current model of therapy is based almost entirely on acute treatment, such as immobilization algorithms and corticosteroid treatment. Although these measures are effective in the short term, they are often limited by serious adverse effects. Other surgical approaches to the problem, including spinal decompression, spinal stability restoration, and bone fragment removal, provide even more, yet still incomplete, functional benefits and are problematized by the presence of adverse effects. Rehabilitative programs aim at improving the quality of life through provision of combined physical and psychological assistance, but their effectiveness is often limited by the high physical and neurological severity of the lesion, as well as by the lack of intervention methods that can trigger significant neural repair.

The therapeutic regimens that are currently available are inadequate in promoting long-term neural repair after spinal cord injury (SCI). Therefore, it is necessary to study other mechanisms and discover new therapeutic targets. The first step is crucial and is associated with clarifying the molecular mechanisms of SCI. One of the pathways involved in endogenous repair, the Sonic Hedgehog (Shh) signaling pathway has become one of the key players in neuroprotection, axonal regeneration, and modulation of inflammation. A thorough knowledge of the molecular processes that regulate the development of the spinal cord and the response to injury are required to find effective therapeutic targets. In this context, Shh signaling, which has both effects in embryonic development and wound healing, has been a consistent subject of scientific interest.

## 2 Mechanisms of Sonic Hedgehog signaling in spinal cord development and injury

### 2.1 Molecular mechanism of Shh signaling

The advances in medicine, especially the discovery of the Sonic Hedgehog (Shh) are considered as an important step forward in the understanding and treatment of spinal cord injury. Shh is an important regulatory agent secreted by the notochord during embryonic development. Recent studies have shown that the Shh signaling pathway plays an important role in neuroinflammation and neural repair after neurological injury, promoting neural regeneration as well as antioxidant and anti-inflammatory effects (Li et al., 2021).

In 1980, Nüsslein-Volhard and Wieschaus (1980) identified a developmental gene in *Drosophila* that, when absent or mutated, causes the *Drosophila* embryo to develop into a hairy mass resembling a hedgehog and named it the Hedgehog gene (Ma et al., 2022). While only a single Hedgehog gene has been identified in primitive invertebrate such as *Drosophila*, three Hedgehog homologs have been identified in complex animals: Sonic Hedgehog (Shh), Desert HH (Dhh) and Indian HH (Ihh). Each of these genes encodes a corresponding protein i.e., SHH, DHH and IHH, called ligands. Among these, Shh ligand has been widely studied which plays an important regulatory role in the development of multiple organs such as neural stem formation, stem cell differentiation, lungs and hair in mammal species (Li et al., 2021).

**FIGURE 1:**
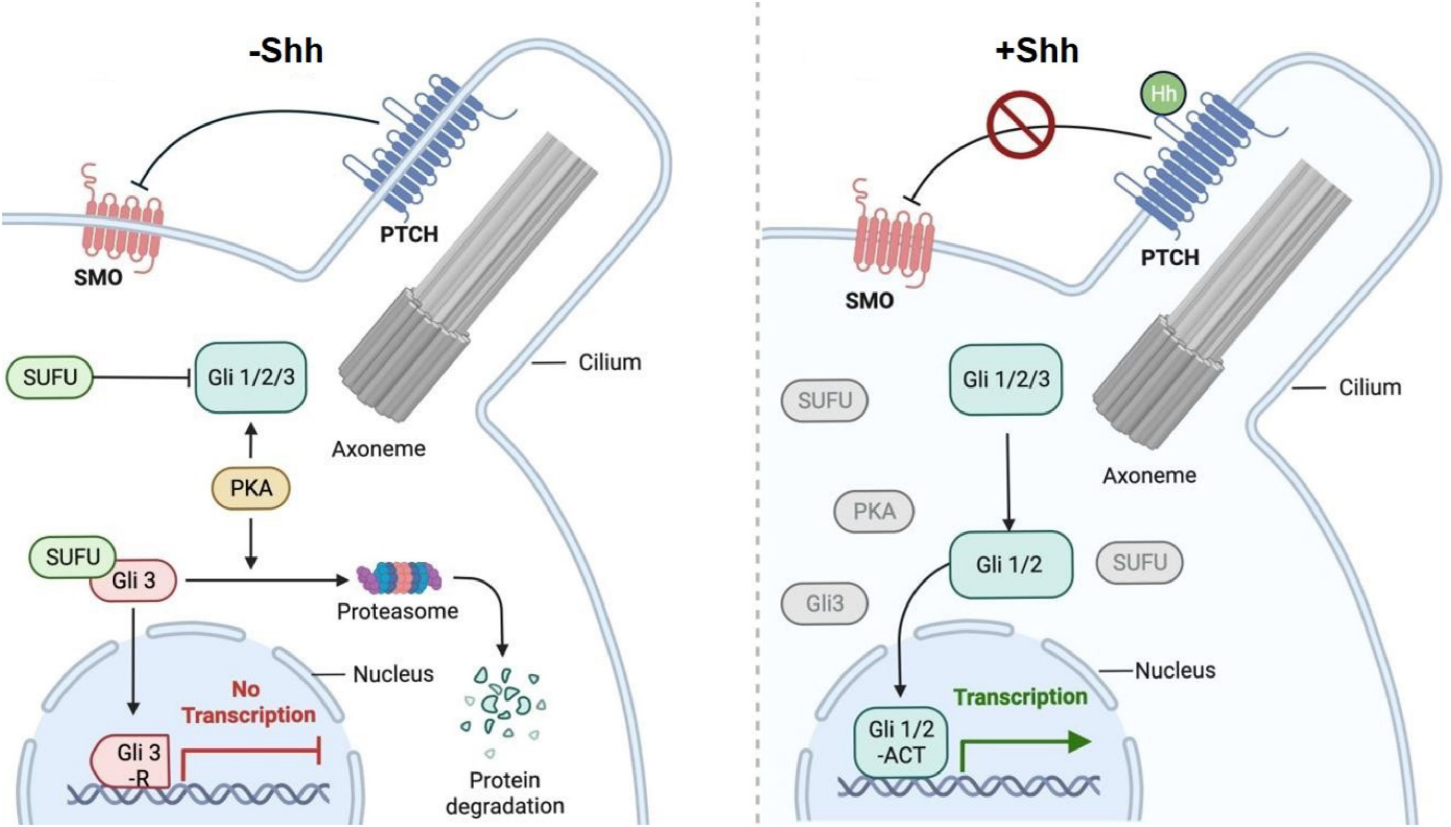
Shh signaling pathway. Patched (Ptch1) inhibits Smoothened (SMO) activity in the presence of Sonic hedgehog (Shh-). Ptch1 inhibits SMO and thus inhibits downstream signaling. Gli protein is phosphorylated by protein kinase A (PKA), leading to its hydrolytic shearing by protein and the formation of the carboxy-terminal truncated repressor Gli 3, which travels to the nucleus and represses Gli-dependent transcription of target genes. With Shh + expression, Shh binds to Ptch1, thereby derepressing SMO, which activates Gli 1/2 of the downstream signaling pathway, transmitting the signal to the nucleus and activating Gli-dependent transcription of the target gene. (Created with BioRender.com)

The Shh signaling pathway is composed of Shh ligands, transmembrane protein receptor complexes Patched (Ptch) and Smoothened (Smo), transcription factors (Gli protein family) and downstream target genes (Li et al., 2021; Figure 1). Shh ligand, produced by secretory cells in multiple organs, is a highly conserved secretory glycoprotein with a relative molecular weight of about 45 kDa and has the ability to catalyze its own processing, allowing it to split catalytically into two secretory polypeptides; Shh-N with a relative molecular weight of 19 kDa and Shh-C with a relative molecular weight of 26 kDa (Mohan et al., 2023). Recent research has found inactive Shh proteins with intact structures, but when the intact structure of Shh proteins was disrupted and split into Shh-N chains, they become biologically active (Qian et al., 2019). Shh-N is responsible for signaling in the pathway. Shh-C incorporates a cholesterol group into the C-terminus of Shh-N by self-shearing, while the N-terminal end of Shh-N is palmitoylated by acyl transfer to the cysteine residue, which in turn becomes an active Shh protein that can act as a probe in the signaling pathway and exert its signaling ability. Hh proteins are released and diffused into the extracellular mesenchyme via the cell membrane by the synergistic action of the membrane transporter protein Dispatched (DISP) and the secreted glycoprotein SCUBE2, which then binds to the membrane protein receptor (Tukachinsky et al., 2012; Creanga et al., 2012).

There are two protein receptors on the membrane of Shh effector cells: Ptched (Ptch) and Smoothened (Smo). Ptch, encoded by the tumor suppressor gene Patched, is a 12-transmembrane protein with both Hh ligand binding and Smo inhibition functions, serving to negatively regulate the Shh signaling pathway. The human Ptch gene has two homologs, ptch1 and Ptch2, with the Ptch1 gene encoding the Ptch1 protein that is the primary receptor for the Hh protein (Ju et al., 2023). Smo is homologous to the G protein-coupled receptor and is responsible for intracellular signal transduction and activation of target genes. In the absence of Hh ligand, Ptch inhibits the function of Smo. In the presence of the Hh ligand, Ptch binds to Hh, lifting the inhibition of Smo by Ptch is lifted and the Hh pathway is activated (Zhang et al., 2011). Ptch1 is a cholesterol transport protein (transporter) and Smo can be activated by cholesterol, while Ptch1 protein either limits the ability of Smo to acquire cholesterol by transporting cholesterol outward from the membrane (Kaushal et al., 2022). Ptch1 as a transporter protein requires conformational changes to complete the transport of cholesterol or its derivatives, while Hedgehog binding prevents Ptch1 from undergoing conformational changes, thus blocking this process and allowing Smo to be activated.

The receptor Smo is encoded by the proto-oncogene Smoothened. The membrane protein Smo is a 7-transmembrane protein that spans both sides of the cell membrane and is encoded by the proto-oncogene. With a molecular weight of approximately 115 kDa, it is a member of the G protein-coupled receptor family and is a key element in the Shh pathway (Wang et al., 2024). When the repressive effect of Ptch on Smo is released, the activated Smo transmits the signal to the cell and activates the intracellular transcription factors, known as the Gli gene family. There are three nuclear transcription factors in vertebrates, Gli1, Gli2 and Gli3, which have different functions (Chai et al., 2021). The activity of Gli proteins determines the transcriptional status of Hh effector genes and all these transcription factors function in the Shh pathway. The human downstream transcription factor Gli gene has three homologs, Glioma associated oncogene homolog (Glil), GLI-Kruppel family member 2 (Gli2) and GLI-Kruppel family member 3 (Gli3), with Glil and Gli2 acting mainly as transcriptional activators and G1i3 as transcriptional repressors and the DNA binding regions of all three transcription factors are highly conserved (Niewiadomski et al., 2019). Glil is a highly transcriptionally active activator of the Hh signaling pathway. When activated, it serves as a key marker for the pathway’s activation (Sananto et al., 2007).

Intracellular Shh signaling relies primarily on sequential inhibitory interactions for its regulatory effects to occur. Cos2, Fu, SuFu and Gli proteins function in the cytoplasm, with Gli predominantly forming a large Cos2 protein complex aggregated on microtubules (Li et al., 2021). When Shh signaling is in an inactive state, Smo located in the cell, interacts directly with the Cos2 protein complex through the C-terminus, while Ptch binds to Smo in the suppressed state. After activation of Shh signaling, Shh binds, Ptch and changes the spatial conformation of Ptch protein. The Ptch/Smo complex then releases Smo from inhibition, allowing it to become highly phosphorylated and translocated intracellularly to activate the downstream effector molecule Gli. Gli then enters the nucleus to induce the expression of target genes such as Wnt (Zhu et al., 2017). During the activation of the Shh signaling pathway, Shh proteins and Smo proteins play a positive regulatory role, while Ptch functions as a negative regulator. The transport process of Smo is related to signaling and depends on the cytoskeleton and microtubule (Jia et al., 2003).

When the organism is under normal, unstimulated conditions, Ptch exerts an inhibitory effect on Smo and the Shh signaling pathway remains inactive. However, when the organism is stimulated or mutated, Shh ligands bind to the membrane protein Ptch, which enter the nucleus to initiate the transcription of target genes, promoting cellular proliferation, differentiation and migration in response to external stimulation or genetic mutation (Smoak et al., 2005; Palma et al., 2005).

### 2.2 Shh in morphogenesis of spinal cord

Sonic Hedgehog plays an extremely important role in the morphogenesis of the spinal cord. Shh is detected during early vertebrate embryonic development and is expressed in midline tissues such as the spinal cord floor plates, notochord and nodes, where it controls the establishment of the left-right and dorsoventral axes of the spinal cord during embryonic development (Watanabe and Nakamura, 2000; Meyer and Roelink, 2003). During neural tube development, Shh from the notochord induces the expression of Shh at the ventral floor plate and slowly spreads to the dorsal side of the spinal cord, resulting in a higher concentration of Shh ventrally than dorsally. These varying concentrations play different roles in inducing the differentiation of neuronal precursor cells and glial precursor cells into neurons or glial cells (Watanabe and Nakamura, 2000; Meyer and Roelink, 2003). At the same time, Shh plays an extremely important role in the transmission of information between the brain and the surrounding tissues.

Studies have shown that Shh is an important chemical elicitor of axon guidance molecules, capable of directing joint neuronal axons located in the developing dorsal part of the spinal cord’s pterygoid plate to be induced by high concentrations of Shh to project to the basal plate (Charron et al., 2003). When the axon reaches the floor, a receptor called Hip is produced on the surface of the axon, which repels the Shh signaling molecule and causes the joint neuron axon to leave the base plate. Subsequently, the combined effect of Shh repulsion and the attraction of Wnt causes the joint neuron axons to form the correct axonal projections, which are finally sorted into different fibers in the spinal cord and projected precisely to the hindbrain, cerebellum and midbrain.

When specific antibodies are used to prevent Shh from functioning properly, the axonal guidance of neurons is disturbed, resulting in a number of abnormalities, such as subplate retention, random forward-backward projections and over-projections (Parra and Zou, 2010). In conclusion, Shh is an important inducible molecule during spinal cord development, capable of regulating the differentiation of neural precursor cells and controlling the formation of the dorsoventral axis of the spinal cord. Also, as an axon guidance molecule with dual functions, it can direct the axons of developing joint neurons in the spinal cord project in the correct direction, ensuring that appropriate connections are established between neurons in different parts of the spinal cord. The detailed understanding of Shh signaling components provides the basis for developing pharmacological strategies aimed at modulating this pathway for therapeutic purposes in SCI.

## 3 Therapeutic modulation of Shh signaling in spinal cord injury

Several drugs have the potential to regulate Shh signaling pathway by modulating its components. These drugs can stimulate or suppress Shh signaling, thereby influencing the normal healing process of SCI. Based on their mode of action, these drugs are classified into agonist and antagonists. An overview of potential drug targeting the Shh signaling pathway with their mechanisms, benefits and research advancements has been given in Table 1.

**TABLE 1:** Overview of potential drugs targeting the Shh pathway.

Drug	Type	Mechanism of action	Model used	Route of delivery	Observed effects	Limitations	Developmental stage	References
SAG (smoothened agonist)	Agonist	Activates SMO protein without Shh ligand	Mouse SCI	Intraperitoneal	Promotes axon growth, neuronal survival and tissue repair	High doses may cause uncontrolled cell division, tumor risk and excessive scarring, sex-specific immune activation (females)	Preclinical	Reimer et al., 2009; Kassoussi et al., 2024
Purmorphamine	Agonist	Directly activates SMO protein	Rat SCI	Systemic	Facilitates neurogenesis, axon regeneration, prevents neuronal apoptosis	Short half-life, frequent administration required	Preclinical	Prajapati et al., 2024
Recombinant Sonic Hedgehog N-terminal fragment (SHH-N)	Ligand	Mimics Shh protein, binds with PTCH1 and prevents inhibition of SMO	Zebrafish, Rat	Intrathecal, hydrogel	Promotes remyelination, reduces neuronal apoptosis	Short half-life, poor tissue penetration, rapid excretion	Preclinical	Gulino et al., 2010; Zhang et al., 2017
Cyclopamine	Antagonist	Binds to SMO, inhibits Gli transcription factors	Mouse SCI	Intrathecal, systemic	Prevents excessive glial scar formation	Impairs regeneration, neurotoxic with high doses	Preclinical	Charron et al., 2003; Parra and Zou, 2010
Vismodegib and Sonidegib	Antagonist	Inhibit SMO activation, silence Gli transcription factors	Cancer study	Oral	Not yet tested in SCI	Systemic toxicity, teratogenicity	FDA approved in cancer	Sekulic et al., 2012

### 3.1 Pharmacological agonists

Agonists play a crucial role in activating Shh signaling pathway by enhancing the activity of Shh, Smo and Gli1. These compounds promote downstream signaling that increases cell proliferation, tissue repair and neuroprotection. In the context of SCI, Shh agonists improve functional recovery by reducing inflammation, preventing neural apoptosis and facilitating the neural regeneration. Some of the Shh agonists with promising results have been discussed below.

Smoothened agonist is a synthetic molecule that plays a crucial role in stimulating Shh pathway. It boosts natural healing process in SCI by promoting axon growth and neuronal survival (Sun et al., 2023). Its binding with SMO activates the stimulation of SMO protein without requiring Shh ligand. Activated SMO proteins controls cell survival, growth and tissue repair by regulating Gli transcription factors (Burton et al., 2022). However, the use of SAG in SCI is limited due to its adverse effects as the high dose of SAG may lead to excessive activation of Shh pathway, resulting uncontrolled cell division. It can enhance the risk for tumor formation and excessive scar formation. Additionally, non-specific delivery of SAG in normal tissues allows the abnormal cell growth (Rahi and Mehan, 2022; Prajapati et al., 2023).

Recent experimental studies have demonstrated that intranasal formulation of SAG provides highest efficacy in body. Efficacy of SAG is not same in males and females (Kassoussi et al., 2024). In females, SAG is responsible for production of pro-inflammatory cytokines which reduces therapeutic potential in spinal cord injury. While in males, the high levels of testosterone counteract production of cytokines. It is responsible for initiating myelin regeneration and proliferation of oligodendrocyte progenitor cells in spinal cord injuries (Kassoussi et al., 2024). Research is ongoing to optimize the dosage of SAG, ensuring the controlled stimulation of the Shh pathway, while preventing uncontrolled cell division. Recently, nanoparticles have been employed for the targeted delivery of SAG to the injury site enhancing its therapeutic effect while minimizing the risk of abnormal cell proliferation in healthy tissues (He et al., 2024).

Purmorphamine is a small-molecule agonist of the Shh signaling pathway that directly activates SMO protein without requiring Shh ligand. This activation triggers the Shh pathway leading to increased activity of Gli1 and Gli2 transcription factors which regulate genes involved in neuronal survival and axonal growth (Gupta et al., 2022). As a result, purmorphamine aids in recovery of SCI by promoting neurogenesis, enhancing axon regeneration and preventing neuronal apoptosis (Prajapati et al., 2024). In spite of high therapeutic potential, purmorphamine has been studied in detail in preclinical conditions. Its fast excretion and relative low bioavailability require its frequent administration, thus limiting its clinical application. To overcome these challenges, researchers are developing chemically modified forms with improved half-life and stability.

Recombinant Shh-N is a synthetic molecule having N-terminal fragment mimics Shh protein resulting Shh pathway activation. It helps the recovery of SCI by promoting remyelination of neurons and reduces neuronal apoptosis. It binds with PTCH1 receptor and prevents the inhibition of SMO. This leads to activation of Gli transcription factors which controls the regulation of genes. Activation of genes regulates remyelination of neurons and neuronal apoptosis (Lamson et al., 2024). The therapeutic potential of Shh-N is still at subclinical level due to its fractional half-life and its scarcity and non-specific penetration of tissue. Its beneficial effect in spinal cord injury requires continuous administration due to its quick elimination in the body. Furthermore, its effective transportation to the point of wound is still challenging, as it is structurally unstable, its molecular weight size is relatively large and the injured tissues have limited permeability. For instance, hydrogels and nanoparticles are being experimented for the specific delivery of Shh-N to injured site which could enhance the therapeutic role of Shh-N in spinal cord injury.

### 3.2 Pharmacological antagonists

Antagonists are the drugs that regulate Shh pathway by suppressing its excessive activation, preventing abnormal cell proliferation and excessive scar formation at the site of injury. By inhibiting key components like SMO or Gli proteins, these drugs help maintain controlled tissue repair, reducing fibrosis and promoting functional recovery (Infante et al., 2015).

Cyclopamine is a plant-derived alkaloid which helps in recovery of spinal cord injury by preventing excessive glial scar formation. It directly binds with the SMO protein and inhibits Gli transcription factors (Zhang et al., 2022). Inhibition of Gli transcription factor suppresses the expression of genes responsible for astrocyte proliferation. Significant reduction in astrocyte proliferation limits the glial scar formation (da Costa Fernandes et al., 2023). However, its use is prohibited because of its potential to completely block Shh pathway. Blockage of Shh pathway slows down the natural healing process of body by promoting neuronal apoptosis. Researchers are currently trying to develop a modified formulation of cyclopamine that selectively blocks Shh pathway specifically at fibrotic areas, minimizing off-target effects and improving its therapeutic potential in controlling excessive scarring.

Vismodegib and Sonidegib are FDA approved drugs which plays potential therapeutic role in SCI by limiting glial scar formation (Nguyen and Cho, 2022). These drugs suppresses Shh signaling pathway by inhibiting the activation of SMO protein which leads to silencing of Gli transcription factors (Bao et al., 2023). The expression of genes responsible for astrocyte proliferation is suppressed by silencing of Gli transcription factors. Reduced astrocyte proliferation prevents the formation of dense scar formation at site of injury (Okuda et al., 2022).

The use of Vismodegib and Sonidegib is limited due to its certain drawbacks. These drugs are not prescribed for long term SCI treatment because of their strong side effects in body. Long term use of these drugs can cause nausea, fatigue, muscle cramps and hair loss (Martos-Cabrera et al., 2024). Additionally, these drugs slow down the natural healing process of body by inhibiting Shh pathway. Optimizing their dose and safe delivery method to the injury site could make these drugs a potential therapeutic drug in treating SCI.

### 3.3 Herbal and alternative modulators

In recent years, many studies have shown that traditional Chinese medicine can improve the recovery of neurological function after SCI. However, no evidence is available to date linking any traditional Chinese medicine to the modulation of the Shh signaling pathway in the context of SCI. However, these compounds have shown neuroprotective effects through other mechanisms. For example, Tetramethylpyrazine (TMP) activates Akt/Nrf2/HO-1 signaling pathway leading to inhibition of ferroptosis and neuroprotection, thus promoting recovery after SCI (Tao et al., 2024). Likewise, sulforaphane (SFN) activates the Nrf2/ARE signaling pathway reducing the oxidative stress and inflammation, thus providing neuroprotection in SCI. Furthermore, Tanshinone IIA (TIIA) has been reported to attenuate inflammation and apoptosis after SCI, possibly via modulation of the Notch signaling pathway.

It was observed in animal models that TMP promoted functional recovery after spinal cord injury in rats by reducing the inflammatory response, inhibiting MMP2, MMP and down-regulating miR-214-3p to attenuate neuronal apoptosis after spinal cord injury (Jia et al., 2021; Fan and Wu, 2017; Hu et al., 2013). A meta-analysis assessed the efficacy of several non-traditional herbs including SFN, TIIA and TMP in reducing inflammation and compared them to a known effective anti-inflammatory agent interleukin-10 (IL-10). The results of the study showed that TMP has a significant effect in reducing inflammation through the upregulation of IL-10, while sulforaphane and TIIA also demonstrated anti-inflammatory effects but were less potent than IL-10 (Koushki et al., 2015). Ding et al. (2022) found that acupuncture combined with moxibustion therapy improve the expression of Shh and Gli-1 after the injured spinal cord of rats. This may be part of the underlying mechanisms of acupuncture combined with moxibustion treatment, including restoration of motor function, protection of neuronal cells and attenuation of apoptosis of nerve cells in rats after SCI.

Though, the relationship of traditional Chinese medicine with Shh in context of SCI recovery has not been evaluated, some evidences linking TMP and SFN to the Shh pathways are available in the context of cervical and lung cancers. TMP has been found to suppress the Shh pathways and inhibit the proliferation, invasiveness and migration of cervical cancer cells. Furthermore, by inhibiting the Shh pathways, it has been found to attenuate sinusoidal angiogenesis in liver fibrosis (Zhao et al., 2017). Similarly, SFN has been reported to inhibit the self-renewal of lung cancer stem cells and proliferation of leukemia stem-like cells through Shh pathway (Wang et al., 2021; Wang et al., 2022).

While these therapeutic agents have demonstrated promising effects in preclinical studies, their clinical translation depends on several factors including efficacy in complex biological environments, safety and delivery challenges.

## 4 Clinical potential and translational applications of Shh-targeted therapies

### 4.1 Role of Shh in spinal cord regeneration

Sonic Hedgehog signaling pathway has progressively been found to be linked with spinal cord injury (SCI), and this factor has led to focus on its involvement in post-injury repair. According to Reimer et al. (2009), the expression of Shh in the adult zebrafish spinal cord was highly increased after SCI, as compared to the uninjured controls. Notably, a Shh pathway-specific inhibitor cyclopamine significantly inhibited Shh expression and thus cell proliferation and neuronal regeneration. These data indicate that high levels of Shh activity are of paramount importance to the motor neuron regeneration. In the same way, Gulino et al. (2010) showed that Shh acts synergistically with the Notch-1 signaling pathway to regulate synapsin-I, which enhances synaptic remodeling and neural regeneration and, therefore, leads to enhanced functional recovery of the damaged spinal cord. Thomas et al. (2014) noted that dual treatment with Shh and neurotrophic factor-3 increased the number of oligodendrocytes and myelin production, which are crucial to the functionality of the axons and remyelination following SCI. Zhang et al. (2017) furthered these observations demonstrating that the PTC1 and PTC2 gene silencing induced Shh signaling cascade during the acute period of SCI, with higher expression of Shh, Smo, and Gli1 related to decreased inflammation, tissue cavitation, and local nerve regeneration. Altogether, these studies highlight the versatile nature of Shh signaling in spinal cord repair, such as its effect on neurogenesis, synaptic plasticity, myelination, and immune regulation. Such mechanistic knowledge makes the Shh pathway a prime molecular target in the design of new therapeutic approaches to SCI, but more work is needed to establish its clinical significance and to maximize its manipulation.

Intrathecal Sonic Hedgehog (Shh) treatment produced neuroprotective effects in rats with severe contusion/compression spinal cord injury (SCI) with reduced neuroinflammation, decreased astrocyte proliferation, and enhanced post-injury locomotor recovery 6 weeks after injury onset (Zhang et al., 2021). Simultaneously, Jia et al. (2021) revealed that bone marrow mesenchymal stem cells-derived exosomes were especially effective in repairing SCI, and Shh signaling was a central molecular mediator of this positive outcome. All together these studies suggest that strong stimulation of Shh signaling pathway after SCI correlates with functional recovery and therefore is a new therapeutic target in SCI treatment. While, several studies suggest that Shh promotes neural regeneration, others raise concern about the potential for excessive astrocyte proliferation leading to glial scarring. This duality underlines the need for precise regulation of pathway activation as beneficial effects may turn maladaptive in prolonged or unregulated contexts. Despite encouraging results in animal models, translating Shh-based therapies into clinical practice requires addressing numerous unresolved challenges and biological complexities.

## 5 Current challenges, research gaps and future prospects

The therapeutic application of Shh signaling in SCI remains constrained by the complexity of the post-injury microenvironment. Following SCI, multiple signaling pathways including Wnt, MAPK, NF-κB, mTOR and Notch are concurrently activated to coordinate processes such as neuroinflammation, cell survival and tissue repair (Ding and Chen, 2023). These pathways exhibit extensive cross-talk and the precise role of Shh signaling varies depending on the cellular context and timing. As such, understanding how Shh interacts with other signaling networks is critical, yet remains incompletely understood.

A major therapeutic barrier is the risk of tumorigenesis associated with excessive or prolonged Shh activation, particularly, when using potent agonists like SAG. Off-target effects in non-neural tissues and variability in immune responses such as pro-inflammatory activation observed in females further complicate treatment generalizability. These concerns highlight the necessity for tightly regulated, localized delivery systems that minimize systemic exposure and reduce oncogenic risk.

Another key challenge is the modulation of chronic inflammation following SCI. Sustained inflammation contributes to secondary damage, glial scarring and inhibition of axonal regeneration. Shh signaling has been shown to influence the inflammatory environment but its dual role in both promoting repair and potentially exacerbating astrocyte proliferation necessitates precise temporal control. The therapeutic benefit of Shh activation is highly context-dependent, requiring an optimal therapeutic window to maximize neuroregeneration while minimizing adverse effects.

Future research must focus on defining this therapeutic window, optimizing dosage and improving delivery strategies such as the use of nanoparticles or hydrogels for targeted release. Emerging approaches combining Shh pathway activation with stem cell therapy or anti-inflammatory agents hold promise. Stem cells may enhance neurogenesis and provide structural support, while anti-inflammatory agents can modulate the microenvironment to favor repair. However, robust preclinical models and longitudinal human studies are needed to assess the safety, efficacy and long-term outcomes of these combinatorial strategies.

Collectively, these challenges underscore the importance of a systems-level approach to SCI repair. Advancing Shh-targeted therapies will depend not only on refining molecular interventions but also on integrating them into broader, multi-modal treatment frameworks.

## 6 Conclusion

Sonic Hedgehog signaling plays a vital role in spinal cord development and regeneration, making it a promising target for therapeutic intervention in spinal cord injury (SCI). Preclinical studies highlight its potential in promoting neuroprotection, remyelination and axonal repair. However, challenges such as tumorigenicity, off-target effects and limited human data hinder clinical translation. Future research should focus on precise delivery systems, combinatory approaches with stem cells or anti-inflammatory agents and well-controlled clinical trials. Advancing these strategies could position Shh modulation as a key component of integrated SCI treatment in the future.
